# A Latent Model for Prioritization of SNPs for Functional Studies

**DOI:** 10.1371/journal.pone.0020764

**Published:** 2011-06-08

**Authors:** Brooke L. Fridley, Ed Iversen, Ya-Yu Tsai, Gregory D. Jenkins, Ellen L. Goode, Thomas A. Sellers

**Affiliations:** 1 Department of Health Sciences Research, Mayo Clinic College of Medicine, Rochester, Minnesota, United States of America; 2 Department of Statistical Science, Duke University, Durham, North Carolina, United States of America; 3 Department of Epidemiology and Genetics, Moffitt Cancer Center, Tampa, Florida, United States of America; University of Leuven, Belgium

## Abstract

One difficult question facing researchers is how to prioritize SNPs detected from genetic association studies for functional studies. Often a list of the top *M* SNPs is determined based on solely the p-value from an association analysis, where *M* is determined by financial/time constraints. For many studies of complex diseases, multiple analyses have been completed and integrating these multiple sets of results may be difficult. One may also wish to incorporate biological knowledge, such as whether the SNP is in the exon of a gene or a regulatory region, into the selection of markers to follow-up. In this manuscript, we propose a Bayesian latent variable model (BLVM) for incorporating “features” about a SNP to estimate a latent “quality score”, with SNPs prioritized based on the posterior probability distribution of the rankings of these quality scores. We illustrate the method using data from an ovarian cancer genome-wide association study (GWAS). In addition to the application of the BLVM to the ovarian GWAS, we applied the BLVM to simulated data which mimics the setting involving the prioritization of markers across multiple GWAS for related diseases/traits. The top ranked SNP by BLVM for the ovarian GWAS, ranked 2^nd^ and 7^th^ based on p-values from analyses of all invasive and invasive serous cases. The top SNP based on serous case analysis p-value (which ranked 197^th^ for invasive case analysis), was ranked 8^th^ based on the posterior probability of being in the top 5 markers (0.13). In summary, the application of the BLVM allows for the systematic integration of multiple SNP “features” for the prioritization of loci for fine-mapping or functional studies, taking into account the uncertainty in ranking.

## Introduction

Many genome-wide association studies of complex diseases and phenotypes have been completed in the last decade [1]. Since these only identify the general locus for the risk allele, rigorous and robust methods are needed to select which chromosomal regions should be prioritized for follow-up fine-mapping and/or functional studies. Often a list of the top *M* SNPs is determined based on the p-value from the association analysis and carried forward into the next stage of the study, where *M* is determined by financial constraints. However, this approach is not optimal as rankings are very variable (i.e., variance in the sampling distribution of rankings can be large) and the “causative” variant may not be at the top of ranked order of SNPs [2], [3]. In addition, for many studies of complex diseases, multiple analyses have been completed (e.g., multiple related diseases/phenotypes or subtypes of disease) and integrating these multiple sets of results may be challenging. One may also wish to incorporate biological knowledge, such as whether the SNP is in the exon of a gene or a regulatory region, into the selection of markers to follow-up.

Alternative approaches, that do not prioritize for follow-up based only on ranked p-values, are based on statistical models in which prior knowledge about the SNP can be incorporated into the association analysis, using hierarchical, mixed, or multi-level models [4], [5], [6], [7], [8], [9]. Chen and Witte [9] describe a mixed model framework for modeling *M* SNPs together where the SNP effects are modeled with both the mean and variance of the multivariate normal distribution depending on prior information. Bayesian analysis of case-control studies using power priors to incorporate historical knowledge was proposed by Cheng and Chen [10], while Lewinger et al [11] proposed a hierarchical Bayes method of weighting single SNP association results in a prior model that incorporates previous knowledge.

In this manuscript, we present a Bayesian latent variable model (BLVM) [12], [13], similar to methods used to rank academic institutions and hospitals [14], for the prioritization of markers for follow-up in future replication or functional studies. The BLVM allows for the incorporation of any type of observed information or “features” about a SNP (e.g., p-value, effect size, functional variant, minor allele frequency, published association in the peer-reviewed literature) into a model in which a latent “quality score” is estimated for each SNP. A drawback of other prioritization/ranking approaches is that they do not incorporate the uncertainty of the ranking into the prioritization [3]. Therefore, we propose the prioritization of SNPs to follow-up based on the posterior distribution of the rankings of the latent SNP quality scores [15]. We illustrate the BLVM for prioritization of SNPs for follow-up using data from an ovarian cancer GWAS of 1815 invasive ovarian cases (1070 invasive serous subtype) and 1900 controls. In addition to the application of the method to the ovarian GWAS, where we do not know the “truth”, we apply the BLVM to simulated data, in which we know the truth. The simulated data mimics the setting in which four independent studies have been conducted for four related diseases/traits (e.g., inflammation-related diseases, cancers involving solid tumors) with the incorporation of where or not the marker is non-synonymous (amino-acid changing) coding into the prioritization.

## Methods

### General Formulation of the Bayesian Latent Variable Model (BLVM)

**Figure 1:**
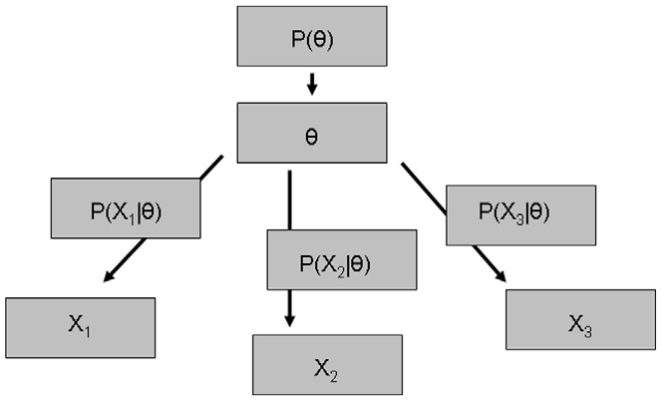
Diagram of Latent Variable Model.

For *K* SNPs in the association analysis, let *θ*_k_, k = 1, …, *K* represent the latent “quality score” for each SNP. We wish to estimate the latent variables *θ*_k_ based on a set of observed features for the SNP, with X_kj_ representing the j^th^ observed feature for the k^th^ SNP. Some possible features may included: −log_10_(p-value), effect size, minor allele frequency (MAF), function of SNP, previously reported SNP or in a pathway or interest. A model is then specified to associate the features with the latent variables. One possible (simple) model is as follows: 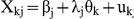, j = 1, …J features, k = 1, …K SNPs where 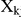 represents the value for the j^th^ continuous feature for the k^th^ SNP, 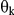 represents the latent “quality score” for SNP k, 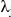 represents the importance of the feature (i.e., how well this feature distinguishes between SNPs), and 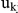 are the random errors and 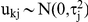 [12], [14]. A graphical depiction of a simplified model is presented in Figure 1. In the case that the feature is binary, there are a few options: a latent probit model could be utilized [16], such that 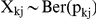 with 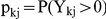 and 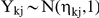; a logistic model 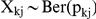 with 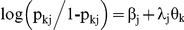 [17], [18].

To complete the model specification, prior distributions are then placed on all parameters in the model. To ensure proper posterior distributions, proper prior distributions, as opposed to improper prior distributions, are placed on all parameters in the model [19]. The prior distributions for the latent scores θ_i_ are typically taken to be independent standard normal distributions, N(0,1). To ensure unique labeling, one can impose strong or constraint priors for a few of the 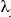 [12]. For example, if it is deemed essential to have a high value of feature to correspond to a high quality score, one could restrict the prior distribution to be a normal distribution censored at 0 (i.e., 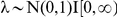). In the case of latent variable models for SNPs, one may also want to model the dependency between the SNPs and their corresponding quality scores by using a prior for 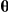 that is multivariate normal, such as 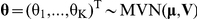 with 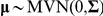 and 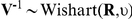, with the matrices 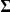 and 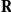 are fixed (e.g., **R** = diagonal matrix consisting of 1). Another choice for modeling the dependency in the SNP quality scores would be to model the dependency between the latent SNP quality scores as a function of LD or spatial distance [8], such as 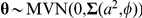, where 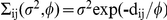 is the covariance between quality scores for SNPs i and j with d_ij_ representing the distance between the two SNPs (e.g., Euclidean distance between the locations of the two SNPs).

### Genome-wide study of ovarian cancer

Ovarian cancer is the fourth leading cause of cancer death among women in the United States. In 2009, it is estimated that 21,550 new cases will be diagnosed in the United States, and 14,600 women will die from the disease [20]. Ovarian cancer risk sharply increases after the age of 40 years and peaks between 65 and 79 years [20]. In the United States, white non-Hispanic women have approximately 40% higher rates of ovarian cancer than Hispanic or African-American women [20]. Most patients are diagnosed with advanced disease because of the lack of an effective screening strategy and the non-specific nature of early signs and symptoms associated with this disease. For the approximate 25% of women who are diagnosed with disease confined to the ovary or ovaries, five-year survival rates are high (75%–90%). For the 75% of women diagnosed with stage III and IV disease, however, the likelihood of long term disease-free survival is low (15%–20%).

The ovarian cases from the US GWAS that will be used for illustration of the latent variable model for ranking SNPs includes four North American studies: (1) FOTS, a population-based study from Ontario 1995–1999, (2) MAYO, a clinic-based study of cases and matched controls in the American upper Midwest 1999–2007, (3) NCOCS, a case-control study covering 48 counties in North Carolina, and (4) TBOCS, a population-based study conducted in Tampa, Florida. The study protocol was approved by the institutional review board at each center (Duke University Institutional Review Board, Mayo Clinic Institutional Review Board, Moffitt Cancer Center Institutional Review Board, Women's College Research Institute Institutional Review Board), and all study participants provided written informed consent. Eligibility for cases is confirmed epithelial ovarian cancer (tubal, primary peritoneal, germ cell, stromal, and unknown histology are excluded) with invasive disease (cases with low malignant potential are excluded). Eligible controls are matched within each study to cases on age, race and residence. All cases and controls were additionally required to have adequate DNA, no prior history colorectal cancer at age less than 50, and no prior history of ovarian, breast, endometrioid cancer; in addition, known non-Caucasian, Jewish, Hispanic, and related participants were excluded. After all samples were genotyped using the Illumina Infinium Human610-Quad BeadChip and quality control had been completed, a total of 1,815 ovarian cancer cases (1,070 invasive serous ovarian cancer) and 1,900 controls were available for association analysis.

Analysis for association of genetic markers with cancer status (all invasive ovarian cancer cases versus controls), along with subtype analysis of invasive serous ovarian cancer cases versus controls, was completed using PLINK software [21]. Results from a randomly selected chromosome (chromosome 20) were utilized to illustrate the use of the latent variable model in prioritizing SNPs for follow-up in functional studies (accounting for the uncertainty in the ranking).

### Five specific latent models applied to ovarian GWAS

Below, we outline five specific latent variable models for the ranking of SNPs which were applied to the ovarian cancer GWAS. The five BLVMs for prioritization of SNPs involve the following SNP “features”: Minor allele frequency (MAF), p-value and odds ratio (OR) from analysis involving all cases and p-value and OR from analysis involving a subset of the cases (i.e., histological subtype). All features were first transformed such that “large” values of the factor would result in a “large” SNP quality score. In addition, transformations for the various features were selected such that they could be modeled using a normal distribution (for speed in computation of the MCMC). It should be noted that additional SNP features could be included in the model, such as, whether or not the SNP is a non-synonymous coding variant or associated with mRNA expression (eSNP) [22], [23]. Likewise, a variety of transformations of the features could also be employed. For our presentation of the BLVM, we chose the following transformations for the SNP features: *f*(p-value) = −logit(p-value), *g*(MAF) = logit(2*MAF), *h*(OR) = log(OR) if OR>1 and log(1/OR) if OR<1. The transformation selected for the odds ratios resulted in making the effects all in same direction (“risk”) with the log transformation allowing *h*(OR) to be modelled with a normal distribution censored at 0. Since MAF is between 0 and 0.5, we double the MAF to get a value that ranged between 0 and 1 for the logit transformation that allowed for modeling *g*(MAF) with a normal distribution. Lastly, we chose to transform the p-values using the minus logit with subsequent modeling of *f*(p-value) with a normal distribution. These transformations also allow use of set constraints on the latent variable model to allow for identifiability of the model parameters, with large values for features (*f*(p-value), *g*(MAF) and *h*(OR)) indicating larger SNP quality scores.

**Model 1** involves J = 5 features including p-values and effect sizes for two analysis along with the minor allele frequency for K SNPs, assuming the J features and K SNP quality scores are independent. First, we specify the likelihood model for the J = 5 features as 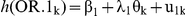 with 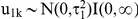, 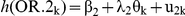 with 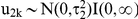, 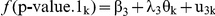 with 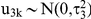, 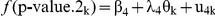 with 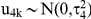, and 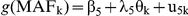 with 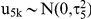, where 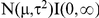 indicates left censored normal distribution at 0 and 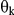 represents the latent “quality score” for SNP k. Next, we specify prior distributions for the parameters in the model. For latent variable models, the direction for the latent variables is arbitrary and without constraints on some of the parameters one can encounter what is referred to as “labeling issues” or “sign changes” [24]. Thus, to ensure unique labeling, we have chosen to impose strong priors on the parameters 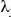 such that the higher the value of the feature the higher the SNP quality score (e.g., SNPs with high values for *f*(p-value) will have a higher quality score than those SNPs with low values for *f*(p-value)). The prior model is specified as 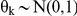 for k = 1,…,K, 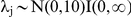, 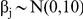 and 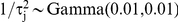 for j = 1,…,5. It should be noted that when only one of the 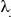 had a strong prior distribution specified to help ensure labeling (i.e., 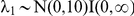), with the remaining parameters having prior distribution unrestricted (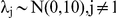), the MCMC failed to converge (as assessed by convergence statistics and trace plots) due to “labeling” issues [25].

The second model we investigated (**Model 2**) was similar to Model 1. However, the odds ratio features were removed leaving only the p-values and MAF features in the model. The rationale for removing the effect sizes from the BLVM was that on observations, it appeared that too much weight might be given to SNPs with very low MAF, as these are the markers that often have the larger effect sizes (but larger standard errors). The third model (**Model 3**) explored was one that was the similar to Model 2 (only p-values and MAF features included) but with the latent quality scores assumed to be dependent and model with the conjugate multivariate Normal – Wishart prior. That is, we model the latent SNP quality scores as 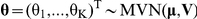 with 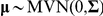 and 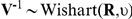, with the matrices 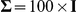, 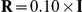 and 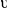 = K where 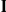 is a K×K identity matrix. In contrast to modeling the dependency in the latent SNP quality scores, in Model 4 we model dependency between the parameters 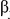. That is, **Model 4** is identical to Model 2 but with the 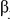
's modeled as 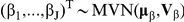 with 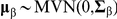 and 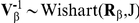, with the matrices 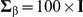, 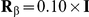 and where 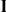 is a J×J identity matrix. The final model investigated (**Model 5**) was again similar to Model 2 but with fewer constraints for identifiability, with only constraints placed on the parameters 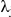 for the p-value features and not the MAF feature.

The BLVM can be fit and parameters estimated within a Markov chain Monte Carlo. For application of the BLVM to prioritization of SNPs, we are mostly concerned with the latent SNP “quality scores”, θ_k_ and not the parameters 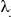 and 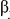. In addition to parameter estimation for θ_k_, we are also concerned with the relative ranking of the SNPs, along with the incorporation of uncertainty in the rankings. For example, we can estimate the probability that a given SNP will be in the top 5 based on the posterior distribution of the rankings to aid in the prioritizing of SNPs for follow-up functional or fine-mapping studies. A benefit of completing the latent variable modeling within a Bayesian formulation is the flexibly of model form and the ability to assess model fit. Various models can be fit and to assess the robustness of the findings. For example, instead of assuming normality of the quality scores θ_k,_ we could assume the scores follow a heavier tailed distribution (e.g., t-distribution).

### Simulated data

To illustrate the use of the BLVM for the prioritization of SNPs from multiple GWAS studies along with the incorporation of functional information for the variants, we simulated 10 replicate sets of results (e.g., p-values for single SNP association) for 100 markers and four disease phenotypes (e.g., ovarian cancer, breast cancer, prostate cancer, pancreatic cancer) for four scenarios. The objective of the application of the BLVM is to determine possible genetic variants relevant with the four phenotypes that should be prioritized for functional studies. In simulating the SNP association p-values, we treated markers 10, 20 and 40 as non-synonymous coding variants, with the remaining markers considered “non-coding” variants. Scenario 1 represents the case in which none of the markers was associated with the phenotypes (e.g., null). The second and third scenarios involving markers being associated with the first two disease phenotypes; marker 10 (coding SNP) associated in scenario 2 and marker 60 (non-coding SNP) associated in scenario 3. The last scenario involved the setting in which marker 60 was associated with all four disease phenotypes. The 100 p-values for the four association studies were simulated, assuming independence, from a Uniform(0,1) distribution for the case of a “null” SNP association and from a Uniform(0, 0.05) distribution for the case of a “non-null” SNP association.

### Specific latent models applied to simulated data

As outlined for the BLVM for the ovarian GWAS, we chose to transform the four p-values for association for each SNP using *f*(p-value) = −logit(p-value). We coded the functional feature of the k^th^ SNP as C_k_ = 1 if coding SNP and C_k_ = 0 if non-coding SNP. The model we applied to the simulated data (**Model 6**) involved J = 5 features for each SNP (p-values for the four traits and function), modeling all SNP features and quality scores as independent. Let 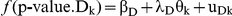 with 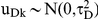, D = 1, 2, 3, 4 and 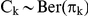 with 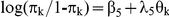 where 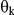 represents the latent “quality score” for SNP k. The prior model is specified as 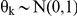 for k = 1,…,100, 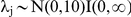, 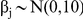 and 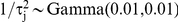 for j = 1,…,5. The amount of weight given to each feature is similar, with each feature effect having a normal distribution with mean 0 and variance 10, censored at 0. To give less to the coding feature, a smaller variance could be used in specifying the coding feature prior (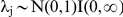), which would results in shrinkage of this effect (and importance) towards zero.

## Results

### Genome-wide study of ovarian cancer

#### Comparison of five latent models

The five different latent variable models outlined above were first assessed using the top 100 SNPs on chromosome 20 from the ovarian GWAS. All five models were fit using the WinBUGS software package [26] by way of the R package BRugs [27]. For each analysis, three independent chains were run, each with 40,000 iterations, with the first 20,000 removed for burn-in of the MCMC. Convergence was checked using trace plots and the 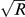 measure discussed by Gelman et al [25].

**Figure 2:**
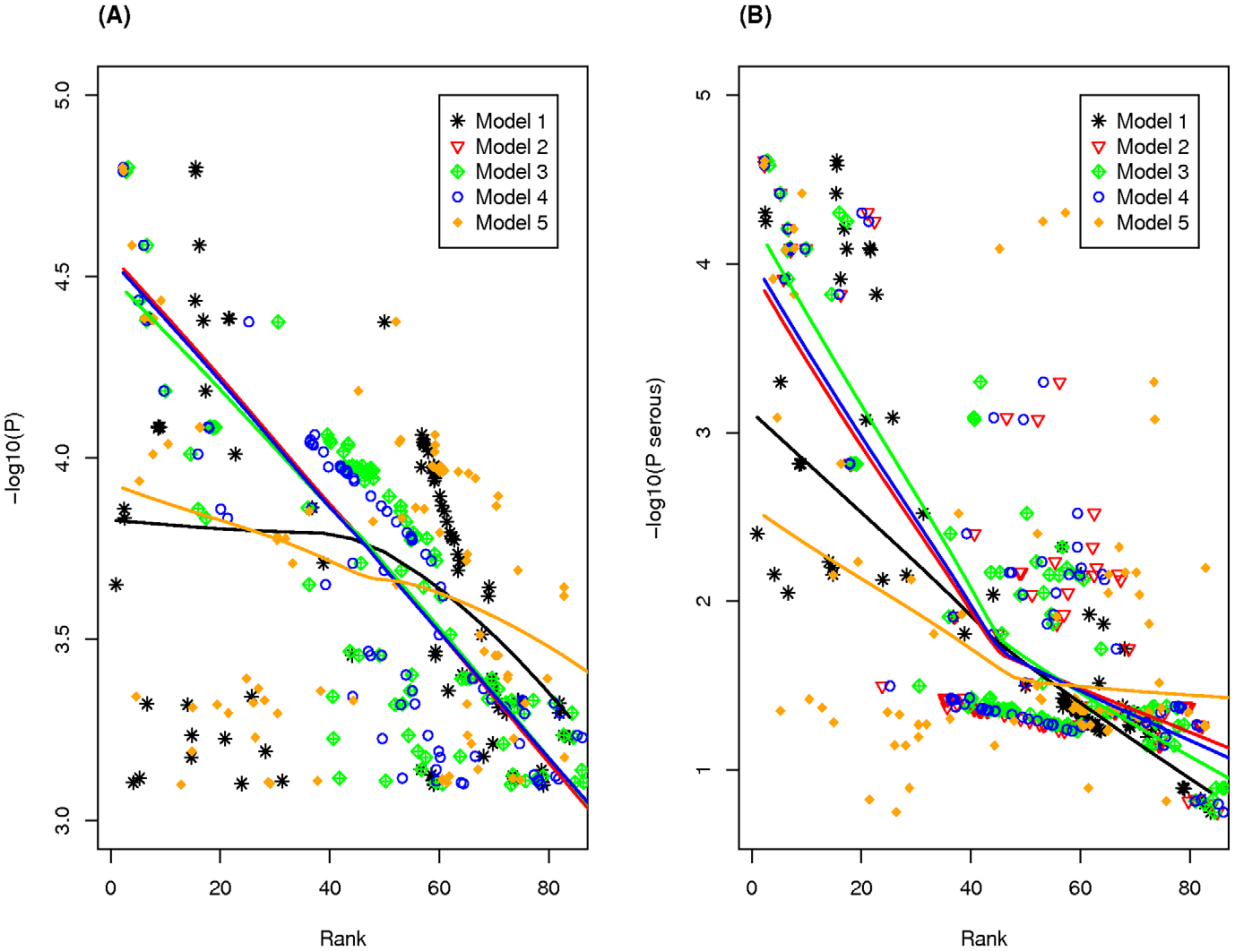
Plot of SNP ranks (mean of posterior distribution of rank) and the −log_10_(p-values) from analyses using (A) all invasive cases or (B) only invasive serous cases for each of the five BLVMs.

**Figure 3:**
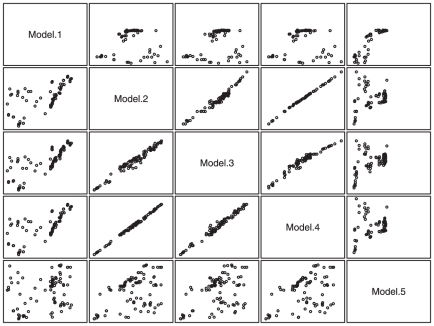
Plots of the mean rank (lower diagonal of sub-plots) and standard deviation in rank (upper diagonal of sub-plots) in the posterior distributions of the rankings from the five BLVMs. The two set of sub-plots are all plotted on the same scale.

Figure 2 shows the relationship between the estimated rank (mean of the posterior distribution for the rank of the latent SNP quality score) and the −log_10_(p-values) for the case-control analysis using all cases (Figure 2A) and the subset of cases with serous histology (Figure 2B). Figure 3 displays the relationship between the ranks (lower diagonal of the scatterplot matrix) and standard deviation (upper diagonal of the scatterplot matrix) in the posterior distributions for the five latent variable models. These figures illustrate the following. First, inclusion of the odds ratios as a feature in the BLVM (Model 1) resulted in SNPs with very low MAF and large effects being ranked in the top SNPs along with rankings from this model inconsistent with (1) rankings based on the other four models and (2) rankings based on the p-values from the case-control association analysis. Second, ranks based on model 2, 3 and 4 are very consistent with similar SD in rankings. In terms of variation in ranks, posterior distributions for rankings of the SNP latent quality score for model 3 had slightly larger variation as compared to models 2 and 4, with no real difference in variation in posterior distribution between models 2 and 4. Lastly, model 5 had lower concordance with model 2, 3 and 4's rankings and p-values from the association analysis, but produce smaller variation in rankings (SD) than models 2, 3 and 4. Based on these results, we opted to use model 2, which is the simplest BLVM model, to estimate the SNP latent quality scores for the top 500 markers from chromosome 20.

#### Ranking of the top 500 SNPs

Based on the results from the ranking of the top 100 SNPs on chromosome 20 using the five BLVM, we next used the simplest model (Model 2) to rank the top 500 markers using the features: p-value from case-control analysis involving all cases, p-value from case-control analysis involving only cases with serous histology, and the MAF for the marker. Table 1 presents the top 40 ranked markers based on the BLVM of 500 markers from chromosome 20, sorted by posterior probability of being in top 5 markers. Results for all 500 markers are presented in Table S1. The markers were ranked based on the mean of the posterior distribution for the latent SNP “quality score”. The top ranked marker (marker 1) from the BLVM had a median rank of 6 and was in the top 5 markers 47% of the time. As the 95% credible interval indicates, there is a large amount of variation in the rank with the interval ranging from 1 to 302. However, marker 1 was ranked 2^nd^ and 7^th^ based on the p-value from the case-control analysis involving all cases and the histological subtype analysis, respectively. Similarly, the second ranked (marker 2) from the BLVM, with median rank of 7 and probability in the top 5 of 0.46, was ranked 1^st^ and 9^th^ based on the analysis of all cases, regardless of histological type, and the cases with serous histological subtype invasive ovarian cancer. In contrast, the top ranked marker (marker 8) based on the subtype analysis p-value (197^th^ based on the all case analysis) ranked in the top 5 markers with probability 0.13 based on the BLVM. The probability of being in the top 5, as opposed to the rank based on the mean of the posterior distribution of the quality score, takes into account the variation in rankings. This can also be seen in the 95% credible intervals for the rankings of the markers.

**Table 1:** Top 40 markers determined from BLVM. The markers are sorted by P(top 5).

Marker	MAF	Invasive		Serous		Rank based on P		Posterior Dist of Ranks	
		P	OR	P	OR	Invasive	Serous	P(top 5)	Median
1	0.139	2.0E-05	0.82	2.0E-05	0.79	2	7	0.47	6
2	0.14	2.0E-05	0.82	3.0E-05	0.79	1	9	0.46	7
3	0.116	3.0E-05	1.22	1.2E-04	1.26	3	24	0.33	15
4	0.13	4.0E-05	0.82	4.0E-05	0.79	4	13	0.27	12
5	0.134	4.0E-05	0.83	6.0E-05	0.8	7	20	0.19	16
6	0.18	4.0E-05	0.84	8.0E-05	0.82	6	23	0.19	17
7	0.18	4.0E-05	0.84	8.0E-05	0.82	5	21	0.19	17
8	0.11	2.0E-03	1.17	1.0E-05	1.32	197	1	0.13	151
9	0.252	4.0E-05	0.86	3.2E-02	0.91	8	286	0.12	52
10	0.126	7.0E-05	1.21	8.0E-05	1.25	9	22	0.09	21
11	0.015	1.4E-04	0.61	5.0E-05	0.51	41	16	0.06	38
12	0.174	1.0E-04	0.85	1.5E-04	0.82	20	26	0.05	29
13	0.015	1.5E-04	1.64	6.0E-05	1.94	43	19	0.05	40
14	0.049	8.0E-05	0.75	1.5E-03	0.76	10	57	0.04	45
15	0.049	8.0E-05	1.32	1.5E-03	1.31	11	58	0.04	45
16	0.049	8.0E-05	0.75	1.5E-03	0.76	13	59	0.04	45
17	0.049	8.0E-05	1.32	1.5E-03	1.32	12	60	0.04	46
18	0.258	9.0E-05	0.87	3.9E-02	0.91	15	301	0.02	74
19	0.258	9.0E-05	1.15	3.8E-02	1.1	16	297	0.02	75
20	0.258	9.0E-05	0.87	3.8E-02	0.91	17	298	0.02	75
21	0.258	9.0E-05	0.87	3.8E-02	0.91	18	300	0.02	75
22	0.259	9.0E-05	0.87	4.3E-02	0.91	14	311	0.02	73
23	0.258	1.0E-04	1.15	4.1E-02	1.09	19	307	0.02	76
24	0.257	1.1E-04	1.15	3.8E-02	1.1	24	296	0.02	79
25	0.258	1.1E-04	1.15	4.4E-02	1.09	21	321	0.02	80
26	0.258	1.1E-04	1.15	4.4E-02	1.09	22	319	0.02	80
27	0.258	1.1E-04	0.87	4.3E-02	0.91	25	318	0.02	81
28	0.258	1.1E-04	1.15	4.4E-02	1.09	23	320	0.02	81
29	0.258	1.1E-04	0.87	4.3E-02	0.91	26	317	0.02	81
30	0.258	1.1E-04	0.87	4.5E-02	0.91	28	329	0.02	81
31	0.258	1.1E-04	1.15	4.4E-02	1.09	30	322	0.02	81
32	0.258	1.1E-04	0.87	4.5E-02	0.91	27	327	0.02	82
33	0.258	1.1E-04	1.15	4.4E-02	1.09	31	323	0.02	82
34	0.147	4.6E-04	1.17	8.2E-04	1.2	67	37	0.01	77
35	0.125	5.9E-04	0.84	8.4E-04	0.81	81	40	0.01	89
36	0.176	1.4E-04	0.85	1.2E-02	0.88	40	218	0.01	78
37	0.002	2.2E-04	0.24	4.0E-03	0.24	53	119	0.01	85
38	0.038	7.6E-04	1.35	5.0E-04	1.44	90	34	0.01	102
39	0.434	3.4E-04	0.89	6.8E-03	0.9	57	186	0.01	96
40	0.434	3.5E-04	0.89	6.8E-03	0.9	59	185	0.01	96

**Figure 4:**
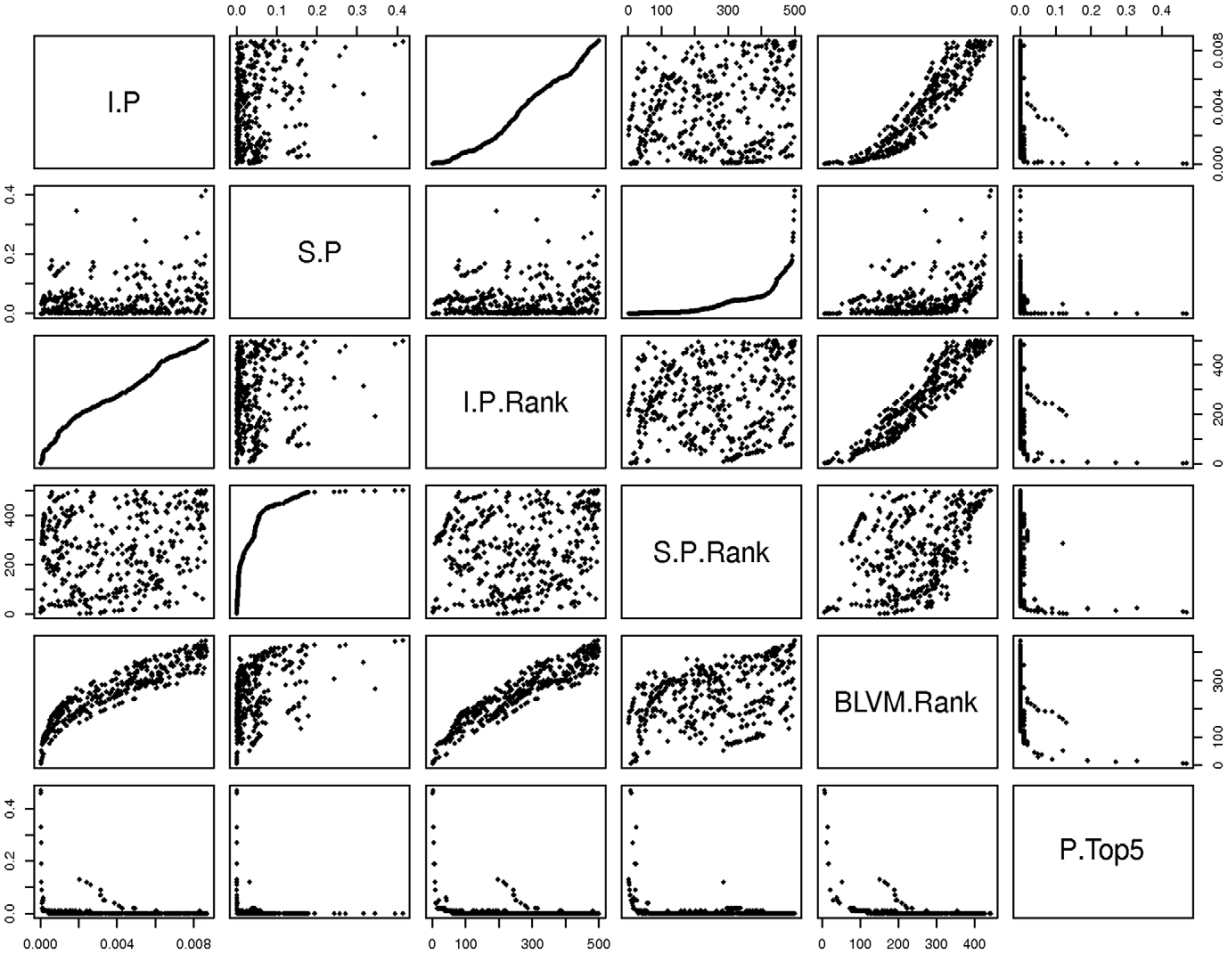
Relationship between SNP association p-values, rankings based on p-values and BLVM and Probability in the top 5 markers. I.P and S.P represent the p-values from the analyses involving all invasive cases or invasive serous cases, respectively; I.P.Rank and S.P.Rank represent the rank of the marker based on the p-values from the analysis involving all invasive cases or invasive serous cases, respectively; BLVM.Rank and P.Top5 represent the median rank and the probability of being in the top 5 markers based on the BLVM.

Figure 4 displays the relationship between the various SNP features and rankings for the 500 markers. As the figure illustrates, the ranking of markers based on the BLVM is related mostly to the p-value from the invasive cases analysis and less so from the results of the invasive serous case analysis and MAF. We also observed that the probability of being in the top 5 markers is highest for markers with small p-values for both invasive and invasive serous analysis is as well as having MAF around 0.10–0.20.

### Simulated Data

The BLVM (Model 6) was applied to each of the 10 simulated datasets, in which the four disease association p-values and the function (coding or non-coding variant) for the 100 SNP markers were included in the latent model. The models were fit using the WinBUGS software package [26] by way of the R package BRugs [27]. For each analysis, three independent chains were run, each with 40,000 iterations, with the first 20,000 removed for burn-in of the MCMC. The mean SNP “quality score” and median rank for Scenarios 2–4 (non-null scenarios) are presented in Table 2, with the mean computed for null and non-null markers along with coding and non-coding markers. Table 3 presents the results for the null scenario (Scenario 1). As the tables illustrate, for Scenario 2 the median ranking for the non-null marker (a functional marker) is in the top 4% of markers in 6 out of 10 simulations, while the average median rank for the null markers was around 50, as expected. When compared to Scenario 3, in which the associated marker is not a coding marker, the median rank for the associated markers is much lower than the ranking from Scenario 2, due to the fact that the non-null marker was not a coding variant. The final scenario in which all four diseases are associated with a non-coding marker, we observe that the ranking for the associated marker improve due to the added information from association with phenotypes 3 and 4. In terms of the null scenario (Scenario 1), the coding markers are ranked slightly higher than the non-coding markers due to BLVM putting some importance on coding variants over non-coding variants (Table 3).

**Table 2:** Summary of simulated p-values and results from analysis using BLVM for Scenarios 2, 3 and 4.

Simulation*	Mean Quality Score		Median Rank		Mean Quality Score		Median Rank	
Scenario 2	Null	Non-Null	Null	Non-Null	Non-Coding	Coding	Non-Coding	Coding
1	−0.029	2.46	50.69	1	−0.052	1.57	51.5	7.3
2	−0.018	1.64	50.95	4	−0.033	1.01	51.5	17.7
3	−0.021	1.81	50.77	3	−0.038	1.12	51.4	13.7
4	−0.023	2.1	51.36	1	−0.037	1.12	51.8	21
5	−0.02	1.73	50.83	4	−0.041	1.24	51.6	11
6	−0.02	2.08	51.11	2	−0.043	1.41	51.8	14
7	−0.016	1.33	50.77	7	−0.027	0.8	51.1	25
8	−0.029	2.81	50.9	1	−0.064	2.05	51.8	4.7
9	−0.022	2.21	51.06	2	−0.04	1.32	51.6	16.3
10	−0.023	2.36	50.82	2	−0.053	1.73	51.6	10.3
Scenario 3								
1	−0.011	0.84	50.82	20	−0.022	0.64	51.3	26.3
2	−0.005	0.19	50.91	51	−0.039	1.15	52.1	11.3
3	−0.009	0.6	50.65	25	−0.033	0.97	51.4	16.7
4	−0.006	0.48	50.28	38	−0.037	1.16	51.3	13
5	−0.025	2.1	51.13	3	−0.004	−0.01	50.7	48.7
6	−0.012	0.74	50.78	26	−0.034	0.95	51.4	21
7	−0.003	0.26	50.77	42	−0.044	1.39	52	7
8	0.002	−0.19	50.2	63	−0.046	1.47	51.6	7.7
9	−0.008	0.66	50.71	23	−0.031	0.96	51.4	19
10	−0.004	0.72	50.92	24	−0.011	0.47	51.1	36.7
Scenario 4								
1	−0.018	1.86	50.76	3	−0.009	0.33	50.8	35
2	−0.017	1.53	50.88	5	−0.026	0.79	51.2	24.7
3	−0.021	1.85	51.25	2	−0.024	0.7	51.4	29
4	−0.018	1.91	50.78	3	−0.021	0.72	51.2	21.3
5	−0.02	1.91	51.19	3	−0.014	0.43	51.1	39.7
6	−0.017	1.54	50.65	6	−0.008	0.19	50.4	42.3
7	−0.023	2.07	51.01	3	−0.005	0.08	50.7	45
8	−0.02	1.75	50.97	4	−0.026	0.77	51.4	22.3
9	−0.038	3.54	51.06	1	−0.004	0.07	50.8	44.3
10	−0.013	1.27	50.48	9	−0.032	1.04	51.1	16.7

*Scenario 2: SNP 10 (coding SNP) simulated to be associated with phenotypes 1 & 2.

Scenario 3: SNP 60 (non-coding SNP) simulated to be associated with phenotypes 1 & 2.

Scenario 4: SNP 60 (non-coding SNP) simulated to be associated with all phenotypes.

**Table 3:** Summary of simulated p-values and results from analysis using BLVM for Scenario 1.

SimulationScenario 1	Mean Quality Score		Median Rank	
	Non-Coding	Coding	Non-Coding	Coding
1	−0.01	0.2	50.75	46.67
2	−0.02	0.7	51.18	27.33
3	−0.03	1.0	51.10	23.33
4	0.00	0.0	50.79	54.00
5	−0.03	0.9	51.32	19.33
6	−0.02	0.5	51.11	35.00
7	−0.02	0.4	50.96	34.00
8	−0.02	0.5	50.93	33.33
9	−0.02	0.6	51.64	30.33
10	−0.04	1.1	51.63	13.67

## Discussion

Over the past few years, numerous GWAS for various complex disease and drug-related phenotypes have been completed, resulting in more than 350 publications and over 1500 SNPs implicated for association with multiple (>80) disease phenotypes or traits [1]. However, the SNPs identified are not necessarily the functional variant, requiring additional research to fine map these putative regions or loci [28] for further biological characterization. Given the extensive efforts involved, it is important to prioritize SNPs for functional studies detected from GWAS. We propose a Bayesian latent variable model (BLVM) to assist in this process.

The BLVM allows researchers to incorporate various “features” about the SNP into the ranking, including results from analysis of multiple phenotypes and prior knowledge, such as whether or not the SNP is a non-synonymous variant or associated with mRNA expression (eSNP) [22], [23]. In addition, the BLVM allows one to quantify the uncertainty in the ranking by estimating the probability that the SNP will be in the top *K* SNPs. The proposed Bayesian latent variable model (BLVM) incorporates these SNP “features” to estimate a latent “quality score”, with SNPs prioritized based on the posterior probability distribution of the quality score rankings. We illustrate the method using data from an ovarian cancer GWAS of 1815 cases (1070 serous subtype) and 1900 controls, and compare the results from the BLVM to the standard ranking of SNPs based on the association p-value. In addition to the application of the BLVM to the ovarian GWAS, we outlined five BLVM models and compared the rankings from these five models. In the end, we opted for the BLVM simplest model for the ranking of SNPs for prioritization for functional studies. Results from the BLVM applied to the ovarian GWAS results for chromosome 20 indicate that if there is only resources to functionally validate a few markers, one should select the two markers with posterior probability of being in the top 5 markers of 0.46. However, for this study, the same two SNPs are selected for follow-up based on the p-value rankings from the analysis of invasive ovarian cancer and controls. In addition, depending on whether the follow-up involves replication of the association, as opposed to completion of functional studies, selection of only one of these two markers is necessary, as they are in high LD.

In addition to the ability of the BLVM to systematically integrate multiple features about the SNPs, the model is flexible in terms of model choice, choice of features to incorporate into the prioritization and weight/importance given to the different features. For example, in the simulated data, we illustrate the use of the BLVM for synthesizing results from multiple genetic association studies conducted on related diseases/traits, as a means for detecting pleiotropic effects (e.g., genetic variants associated with multiple traits). In the application of the BLVM to the simulated data, we also incorporated information regarding whether or not the marker was a coding SNP. The results showed how the inclusion of knowledge about the “functional” aspect of the SNP impacted the results, along with the effect of having all four traits associated with the marker, as compared to only two traits. The application of the BLVM to both the ovarian GWAS and the simulated data, further illustrates the flexibly in model choice and which features to include in the model. For instance, imputation of untyped markers for association analysis in GWAS is becoming a commonly used analysis technique [29], [30], [31]. However, researchers may wish to prioritize observed SNPs over imputed SNPs. This information, or feature, can be included in the model such that SNPs genotyped will be given more weight than SNPs imputed based on a reference panel (e.g., HapMap).

Lastly, sensitivity analysis is possible (and recommended) to assess the impact of modeling choice on the results, as illustrated with the comparison of the five BLVM and the ovarian cancer GWAS. Currently, there is a limitation on the number of markers one can model with BLVM, due to the computational nature of the Bayesian model (i.e., only a few thousand SNPs). Thus, following the genome-wide analysis, a couple thousand markers can be selected (possibly based on univariate or multi-locus p-values or q-values) for which BLVM can be applied using SNP “features” the investigator feels are important in the prioritization, to determine which markers to carry forward into follow-up studies. Another possible approach to reduce the model space would be to remove SNPs in high LD prior to analysis using the BLVM. However, as this approach might be acceptable for follow-up studies involving replication, it might not be an appropriate approach for selecting SNPs for functional studies as one could be removing functional variants in high LD with non-functional variants. Future work is needed to determine the optimal approach to deal with markers in high LD and algorithms to speed up the computation time of the BLVM. In summary, the BLVM is a flexible model that allows for the systematic integration of multiple SNP features, along with the ability to assess the uncertainty in the ranking, for the prioritization of markers for future functional studies.

## Supporting Information

Table S1 **Results for 500 markers from Chromosome 20 with markers sorted by Posterior probability of being in top 5 markers.**
(XLS)
